# A neural perspective on when and why trait greed comes at the expense of others

**DOI:** 10.1038/s41598-019-47372-5

**Published:** 2019-07-29

**Authors:** Patrick Mussel, Johannes Hewig

**Affiliations:** 1 Freie Universität Berlin, Division Personality Psychology and Psychological Assessment, Habelschwerdter Allee 45, 14195 Berlin, Germany; 2 Julius Maximilians University Würzburg, Department of Psychology I, Differential Psychology, Personality Psychology, and Psychological Diagnostics, Marcusstr. 9-11, 97070 Würzburg, Germany

**Keywords:** Psychology, Human behaviour

## Abstract

Depending on the point of view, conceptions of greed range from being a desirable and inevitable feature of a well-regulated, well-balanced economy to the root of all evil - radix omnium malorum avaritia (Tim 6.10). Regarding the latter, it has been proposed that greedy individuals strive for obtaining desired goods at all costs. Here, we show that trait greed predicts selfish economic decisions that come at the expense of others in a resource dilemma. This effect was amplified when individuals strived for obtaining real money, as compared to points, and when their revenue was at the expense of another person, as compared to a computer. On the neural level, we show that individuals high, compared to low in trait greed showed a characteristic signature in the EEG, a reduced P3 effect to positive, compared to negative feedback, indicating that they may have a lack of sensitivity to adjust behavior according to positive and negative stimuli from the environment. Brain-behavior relations further confirmed this lack of sensitivity to behavior adjustment as a potential underlying neuro-cognitive mechanism which explains selfish and reckless behavior that may come at the expense of others.

## Introduction

The dramatic events of the financial crisis in the years 2008 and 2009 have sparked a vivid line of research aiming at a better understanding regarding the definition, measurement, related behavior and neural correlates of the construct of trait greed. At its heart, greed can be defined as an excessive desire for more^1^, thus emphasizing a state of insatiability, associated with the striving for obtaining desired goods. As opposed to mere accumulation, greed may be characterized by hazarding potentially negative consequences that result from one’s own actions, an excessive desire for more at all costs that may be at the expense of others^2^. Trait greed shares this latter aspect with other dark traits like psychopathy and machiavellianism, but is unique with regards to the aspect of striving for more^3,4^. Trait greed has been found to predict several theoretically related behavioral criteria. Among others, trait greed predicted risk-taking; the amount of money allocated to oneself in Dictator and Ultimatum games; self-reported greedy behavior; positive emotional reactions to monetary wins and negative emotional reactions to monetary losses; self-reported risky money investment, immoral behavior, spending more, saving less often, and having debt more often^2,5–9^. However, much less is known about moderating variables of trait greed-behavior relations. Additionally, neurocognitive approaches have just began to investigate underlying cognitive mechanisms of trait greed^5^.

In the present study, we investigate the influence of trait greed in a resource dilemma^10^. Resource dilemmas are characterized by a decision between a selfish choice, which maximizes personal gains at the expense of others, and a cooperative choice, which maximizes the overall benefit for all players^11,12^. Accordingly, selfish choices (sometimes even labeled as greedy choices) share central elements with definitions of greed. Thus, we predicted that individuals with higher, compared to lower levels in trait greed would show more selfish behavior.

Additionally, we investigated two potential moderators of the relation between trait greed and selfish behavior in the resource dilemma. Our first moderator is stakes, that is whether decisions are made with real money or points. The concept of money is a central element of scientific^13^ as well as lay conceptions^14^ of greed. This notion is also supported by empirical evidence. For example, measures of greed have been found to be highly correlated with materialism^6,15^. Thus, individuals with high compared to low levels of greed will especially strive for obtaining real money, as compared to points. We predicted an interaction between trait greed and stakes, which corresponds to the hypothesis that the effect of trait greed on selfish behavior in the resource dilemma will be stronger when playing for real money, as compared to points.

Our second moderator, partner, relates to the interaction partner. As outlined above, greed can be defined as the desire to get more at all costs^2^; thus, greedy individuals might strive for personal gains even if their striving may be at the expense of others^4^. In this regard, greed has also been associated with excessively self-interested behavior that goes against prevailing norms, and as unfair behavior, harming the rules of competition^13^. In line with these arguments, greed has been found to be correlated with psychopathy, especially with the factor meanness^2^. Contrary, for individuals with low levels of greed the prospect of harming another person might refrain them from taking a personal enrichment^16^. We predicted an interaction between trait greed and partner, which corresponds to the hypothesis that the effect of greed on selfish behavior in the resource dilemma will be stronger when playing against another person that is personally known, as compared to a computer.

Investigating neural correlates of personality is at the core of current attempts to arrive at a more fine-grained understanding of personality and its underlying mechanisms^17^. It allows for an additional level of analysis and has the potential of unveiling potential mechanisms that underly personality traits and behavioral effects. Here, we investigate neural responses to the decision of the partner. Same as the participant, the partner has also the choice to either stick to a pre-defined agreement and, thus, make a fair decision, or to defect and, thus, make a selfish decision at the expense of his or her partner (i.e., the participant). Therefore, the decision of the partner can be interpreted as feedback that indicates a favorable vs. an unfavorable outcome for the participant. Such feedback stimuli have been shown to elicit two prominent components, the feedback related negativity^18^ and the P3^19^ in the EEG. The FRN is a component with a maximum at fronto-central electrode positions approximately 200 to 350 ms after stimulus onset that shows a stronger negative deflection after negative, compared to positive stimuli. The FRN has been interpreted within the framework of reinforcement learning theory^20^, which posits that individuals have expectations regarding upcoming events in the future, and that deviations from these expectations are used to learn from experience and, subsequently, adapt behavior. Specifically, events that are “worse than expected”, such as punishment or absence of reward, evoke a negative temporal difference error, whereas events that are “better than expected”, such as reward, evoke a positive temporal difference error and lead to reward positivity^21^. The negative temporal difference error has been related to a phasic decrease in dopaminergic signaling in basal ganglia, followed by a disinhibition of apical dendrites of the motor neurons of the anterior cingulate cortex, which elicits the FRN^22^.

The P3 component typically peaks between 300 and 600 ms and has a positive maximum over parietal electrode sides^19^. It has been found to be sensitive to the magnitude of reward^23^, to the motivational relevance of stimuli^24^ and, according to more recent studies, also to the valence of the reward, with more positive amplitudes for positive compared to negative stimuli^25,26^. Processes reflected by the P3 have been interpreted as contributing to behavioral adjustment to stimuli from the environment^27,28^. The neural source of the P3 component is less well known and is probably distributed over different regions of the cortex, most likely including the temporal-parietal-junction and the locus coeruleus-norepinephrine system^29^. According to prior research, we expected an altered neural response to positive and negative feedback stimuli for individuals high and low on trait greed^5^.

## Results and Discussion

**Figure 1:**
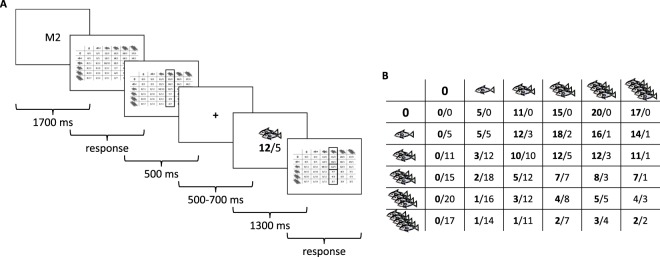
(**A**) Task line and (**B**) an example of the pay-off matrix for the resource dilemma.

In our study, participants played a resource dilemma in which they jointly cultivated a fish farm with a partner (see Fig. 1 and Method). On arrival, they met their partner in person with whom they would play part of the game. They were told that their partner was also a participant. However, the partner was a gender-matched confederate of the experimenter. Participants played four blocks (according to a 2 * 2 design, see Method) with 36 trials each. On each trial, they had to make a decision about how many units of fish (between 0 and 5) to take. The revenue depended on the decision of both the participant and the partner. For a medium decision (two units of fish) of both partners, the overall revenue was maximal. If the participant (but not the partner) took more than two units, the participant would increase his and, at the same time, decrease the revenue of the partner (defecting). However, if his partner also took more than two units, the revenue of both partners would decrease^30^.

On average, participants took 3.0 units of fish (SD = 0.6). We measured trait greed by the GR€€D-scale^2^, a one-dimensional 12-item self-report personality measure (α = 0.90; ω_h_ = 0.93). An example item is “I would stop at nothing to get what I want.”. We found a main effect for trait greed on selfish behavior in the resource game (F = 10.6; p = 0.002; ω^2^ = 0.14). In line with our hypothesis, individuals with higher levels of trait greed took more units of fish from the common resource compared to individuals with lower levels of trait greed.

The ANOVA for the number of units of fish taken by the participants revealed a main effect of stakes (F = 8.5; p = 0.01; ω^2^ = 0.11) indicating that participants took more fish when playing for money, compared to points. This main effect was qualified by an interaction between stakes and partner (F = 13.2; p < 0.001; ω^2^ = 0.17). Post-hoc test showed that the effect of stakes was only observed when participants played against a computer, but not against their partner.

**Figure 2:**
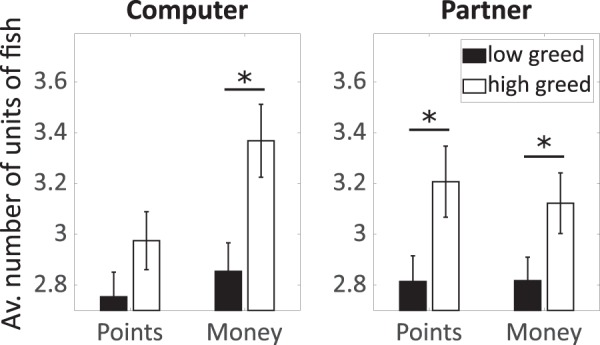
Three-way interaction for the number of units of fish taken by the participant predicted by the factors stakes, partner, and trait greed (according to a median split, for illustration purpose). *Significant effects of trait greed within the levels of stakes and partner.

Regarding the moderating effect of trait greed, neither the interaction between trait greed and partner (F = 0.1; p = 0.80) nor between trait greed and stakes (F = 0.7; p = 0.40) was significant. However, we found a significant three-way interaction between stakes, partner, and trait greed (F = 8.4; p < 0.001; ω^2^ = 0.11, see Fig. 2). Post-hoc tests revealed that individuals high compared to low on trait greed took more units of fish when playing for real money against a computer (p = 0.001), when playing for points against a partner (p = 0.001), and when playing for real money against a partner (p = 0.001), but not when playing for points against a computer (*p* = 0.15). Thus, we found the expected moderating effect for stakes, i.e. the influence of greed was stronger when playing for real money than for points, but only when playing against a computer (rather than against a person). Likewise, we found the expected moderating effect for partner, i.e. the influence of greed was stronger when playing against a real person rather than against a computer, but only when playing for points (rather than for real money). We can therefore conclude that there is partial evidence for the notion that money activates greed, as there is partial evidence for the notion that greed especially manifests when corresponding behavior is at the expense of another person. However, for the combination of the two moderating variables – i.e. when playing for real money against a person – the influence of the two variables did not add up. Rather, there was even a (non-significant) tendency for a reverse effect. One explanation is that the relation between trait greed and selfish behavior already reached a maximum when either of the two moderator variables activated the trait greed-behavior relation (playing for money OR playing against another person^31^); the combination of the two would thus have no further effect, which would correspond to a ceiling effect. Additionally, it can be speculated that there is a certain point at which situational cues activate mechanisms which run counter to greed, such as empathy, care, or fairness concerns. In our case, taking real money from a person that sits in the room next door and whom the person just got to known might be such a situation, which thus overrides the effects of greed. Note, however, that important real-life situations are often characterized by only one of the two variables, such as a hedge fund manager or investment banker making investments on huge amounts of real money in a rather anonymous setting, i.e. for individuals whom he or she does not know and will never get to know.

**Figure 3:**
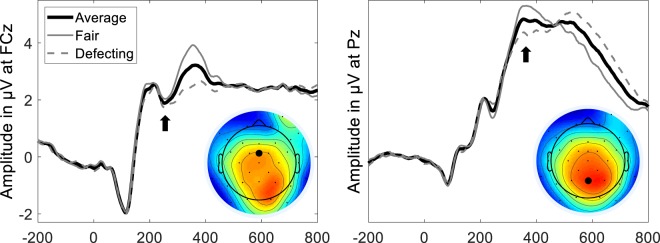
Grand average following the feedback (fair: two units; defecting: 3, 4, or 5 units of fish taken by the partner), at electrode FCz (left) and Pz (right). The topoplot shows the absolute amplitude (+/−16 ms) at 256 ms (left) and 360 ms (right), respectively.

While participants performed the resource dilemma, EEG was recorded from 31 scalp sites (methods). We estimated the FRN as minimum amplitude at FCz between 200 and 350 ms and the P3 as maximal amplitude between 300 and 600 ms and extracted the average amplitude of +/−16 ms around the peak, respectively (see Fig. 3). To investigate neural correlates of decision-making, we ran a three-factorial analysis of variance with the factors stakes, partner and feedback, the co-variate trait greed, and the amplitude of the FRN and the P3, respectively, as dependent variable. For the FRN, to our surprise, we did not find a main effect for feedback (*F* = 3.3; *p* = 0.08). A significant interaction between partner and feedback (*F* = 25.4; *p* < 0.001; ω^2^ = 0.29) indicated that when playing against a partner, but not against the computer, we found the expected FRN-effect: Stronger negative amplitudes for negative (i.e. 3 or more units of fish by the partner) feedback compared to positive (i.e. 2 units of fish by the partner) feedback. This interaction was further qualified by stakes (*F* = 21; *p* < 0.001; ω^2^ = 0.25). Post hoc effects indicated that the expected FRN-effect was only observed when playing against a partner for points. No effects were found for trait greed. We assume that our feedback stimulus, consisting of multiple elements, was too complex to elicit neural correlates that can be observed as early as the FRN (see Liu and Gehring^32^, for similar results).

For the P3, we found a main effect for feedback (*F* = 25; *p* < 0.001; ω^2^ = 0.29). In line with recent findings^25,26^, positive feedback elicited stronger positive amplitudes, compared to negative feedback. Interestingly, we found a significant interaction between trait greed and feedback (*F* = 10.5; *p* = 0.002; ω^2^ = 0.14): The feedback-effect was found for individuals scoring low (p = 0.005) but not high (p = 0.70) in trait greed. This interaction was not qualified by any higher order interactions. A reduced feedback-effect in greedy compared to non-greedy individuals has previously been found for the FRN in a risk-taking task and may be interpreted as a lack of sensitivity to adjust behavior according to positive and negative stimuli^5^. Presumably, this lack of sensitivity might be an explaining cognitive mechanism for a reckless and callous behavior of greedy individuals, namely striving for obtaining personal goals even if it comes at the expense of others. In this regard, note that Dikman and Allen^33^ found a reduced feedback-effect in the FRN for individuals high compared to low in psychopathy, mirroring that greed and psychopathy might share a common cognitive mechanism. This interpretation is in line with both lay conceptions of the greedy psychopath that has been used to characterize agents operating in investment departments of financial firms or stock exchanges as well as with psychometric research indicating a high construct overlap between greed and psychopathy^2^.

**Figure 4:**
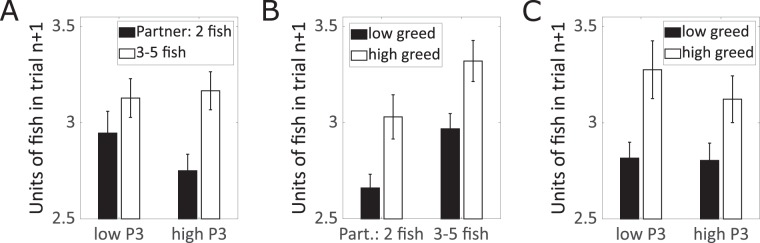
(**A**) Prediction of the number of units of fish taken by the participant in trial n + 1 according to the number of units of fish taken by the partner in trial n and the P3 amplitude in response to the latter in trial n (according to a median split, for illustration purpose). (**B**) As A, but according to the number of units of fish taken by the partner in trial n and trait greed (according to a median split, for illustration purpose). (**C**) As (**A,B**), but according to the P3 amplitude in response to the number of units of fish taken by the partner in trial n and trait greed (both according to a median split, for illustration purpose).

*Finally*, we performed brain-behavior analyses to investigate whether cognitive processes that we observed with regard to the altered P3 response to the decision of the partner would predict subsequent decision-making on a trial-by-trial level using a mixed model where the number of units of fish in trial n + 1 served as dependent variable. First, we found a huge effect of *feedback* (F = 238; p < 0.001; ω^2^ = 0.80): Participants took more units of fish in trial n + 1 if the partner took 3, 4, or 5 fish in trial n (M = 3.15 unit of fish), compared to 2 fish (M = 2.85 unit of fish), which might be interpreted as a revanche effect. Regarding the potential brain-behavior relations, we found a significant interaction between *feedback* and P3 amplitude (F = 11.9; p < 0.001; ω^2^ = 0.16). As illustrated in Fig. 4A, high compared to low P3-amplitudes predicted a stronger revanche-effect, which is in line with theories of the P3 as a component reflecting cognitive processes related to behavioral adjustment^27,28,34^.

Regarding trait greed, we replicated the above-mentioned main effect on selfish decision making (F = 4.4, p = 0.04; ω^2^ = 0.06). An interaction between trait greed and feedback (F = 4.4, p = 0.04; ω^2^ = 0.05) indicated that the effect of trait greed was stronger after positive, compared to negative feedback (see Fig. 4B), which might indicate that after positive feedback, individuals low on trait greed show a tendency to respond to a sign of cooperation in terms of reciprocal fairness, whereas individuals high on trait greed keep showing selfish behavior, rather than adjusting their behavior. Additionally, the effect of trait greed on selfish behavior was also moderated by P3 amplitude (F = 6.2, p = 0.01; ω^2^ = 0.08). The effect of trait greed on selfish behavior was stronger on trials with a lower, compared to higher P3 amplitude (see Fig. 4C). Given the interpretation of the P3 amplitude as mirroring processes according to behavioral adaption, these results are in line with the idea that the effect of trait greed on selfish behavior is mediated by a lack of sensitivity to adjust behavior in response to positive and negative stimuli from the environment.

To sum up, our study provides evidence regarding the influence of trait greed on decision-making in a paradigm in which selfish behavior comes at the expense of others. We found evidence for two moderating variables of this relation which are theoretically related with trait greed: Stakes, that is playing for real money compared to points, and partner, that is playing against a real person compared to a computer. Neural correlates suggest a potential cognitive mechanism underlying these individual differences, namely a lack of behavioral adjustment to positive and negative stimuli from the environment.

## Method

### Participants

Fifty-nine participants were recruited from a paid research participation system (“sona”), postings in the psychology department, and postings over the internet. Participants were between 17 and 56 years old (on average 24.3 years, SD = 5.6), 42 participants were female. They received 12 Euro for participation. The study took about 2 hours. Written informed consent was obtained for all participants. For our sample size, α = 0.05 and a medium effect of r = 0.31, the statistical power to discover an effect if it exists in the population is (1-β) = 0.80. According to the literature, the effect of greed on the FRN effect is assumed to be 0.49^5^. The moderating effect of stakes and partner on decision-making has thus far not been investigated. According to our sample size, a medium effect^35^, a medium effect will be detected with (1-ß) = 80%.

### Task and procedure

Participants played a resource dilemma game^10^. On arrival, the participant and his/her partner were introduced and subsequently filled out informed consent. Next, the participants were told that their partner would play in a separate room, and the confederate left. Actually, the partners’ responses were predefined to control for level of fairness. The experimenter then mounted the EEG, see below.

Participants played four blocks of the resource dilemma with 36 trials each. On each trial, the payoff matrix was first announced (e.g. “M2” for matrix number 2) and then displayed in form of a six times six payoff matrix (see Fig. 1). The columns of the matrix contained the number of units of fish taken by the participant, the rows those by the partner (both ranging from 0 to 5 units of fish).

We used three different payoff matrices differentially encouraging defecting and cooperative behavior. Depending on the payoff matrix, cooperative behavior vs. defective behavior was differently attractive. We used a linear model to compute the payoff for each combination of decisions made by the participant (p1) and the partner (p2) according to:

$$outcome\,p1=\frac{{(unitstakenbyp1)}^{n}}{{(unitstakenbyp1)}^{n}+{(unitstakenbyp2)}^{n}}\times outcome\,(p1+p2)$$

and used three values of n for the three matrices^30,36^: n = 1.2 (encouraging cooperative and discouraging defecting behavior), n = 2 (equally encouraging cooperative and defecting behavior), and n = 3 (encouraging defecting and discouraging cooperative behavior). The payoff matrix for n = 2 is depicted in Fig. 1B.

Based on the payoff matrix, participants made their choice on how many units of fish to take their choice was subsequently briefly highlighted. Next, a fixation cross was displayed for 600 ms with a jitter of +/−100 ms, followed by the feedback. The feedback stimulus consisted of the number of units of fish taken by the partner, along with the corresponding revenue of the participant and the partner. Across the 36 trials of each block, the partner made 18 fair (i.e. 2 units of fish) and 18 defecting (i.e. more than two units of fish) decisions. To enhance the plausibility of the predefined decisions of the partner, the unfair decisions included 9 decisions with 3 units of fish; 6 decisions with 4 units of fish; and 3 decisions with 5 units of fish. According to a pre-study with 71 participants, we found that participants took on average 2.6 (SD = 0.62) units of fish from the common resource. Thus, our experimental manipulation controlled on one hand for an equal number of fair and defecting decisions while on the other hand reflected a realistic pattern of results.

The four blocks correspond to four experimental conditions according to a two times two design with the factors partner (person versus computer) and stakes (money versus points). In the condition person, participants were instructed that they played against the partner that they just got to know in person, whereas in the condition computer, participants were instructed that they played against the computer. In the condition money, participants were told that they played for real money, and that the amount of money obtained during the game would be paid to them after the experiment. In the condition points, they were instructed that they played for points.

After the resource dilemma game, participants filled out the GR€€D scale^2^. The GR€€D-scale is a one-dimensional 12-item self-report personality measure. In the present sample, the measure had good reliability (α = 0.90; ω_h_ = 0.93). At the end of the experiment, participants were debriefed. Due to the deception with regards to the partner, all participants were paid the maximum amount of money that could be won during the game to keep frustration about the deception as low as possible.

All methods were carried out in accordance with the approved guidelines of the Julius Maximilians University Würzburg, and all experimental protocols were approved by its ethic committee.

### EEG Recording and Quantification

While participants performed the resource dilemma, EEG (analog bandpass: 0.1–80 Hz, sampling rate: 250 Hz) was recorded from 31 scalp sites according to the 10–20 system, using Ag/AgCl electrodes and a BrainAmpDC amplifier (Brain Products GmbH, Gilching, Germany). Impedances were kept below 10 kΩ and electrodes were referenced to the vertex (Cz). For detection of blinks and eye-movements the vertical electrooculogram (EOG) was recorded. Data were processed offline, using MATLAB R2017b (MathWorks, Natick, MA) and the Toolbox EEGLAB 14.1.1^37^. First, data were filtered, using a 25 Hz low-pass filter. Subsequently, the EEG was segmented into feedback-locked epochs of 1300 ms (−300 to 1000 ms), baseline-corrected (−200 to 0 ms) and re-referenced to the average across electrodes^38^. Noisy channels (2 channels across all participants) were substituted using interpolation according to a visual inspection of the variance across time pointes. For artifact rejection, trials in which the amplitude exceeded the criterion of absolute amplitude of 300 μV within a segment or the joint probability of 4 standard deviations (joint probability of an electrode’s activity in a segment given that same electrode’s activity in all other segments or joint probability of an electrode’s activity in a segment given all other electrodes’ activities for that same segment) were excluded from further analyses (7.5% of the trials)^39^. At least 10 artifact-free trials were available per participant and condition (M = 16.6). Next, we used ICA decomposition for the detection of eye blink and movement artefacts. Components representing artifacts were detected using the toolbox MARA^40^ and were subsequently removed from the dataset. Finally, data were averaged for each participant and each of eight conditions, defined by the three factors *partner* (person versus computer), *stakes* (money versus points), and *decision of the partner* (fair, i.e. 2 fish, vs. defecting, i.e. more than 2 units of fish).

To quantify the FRN, we estimated the minimum amplitude between 200 and 350 ms at FCz and extracted the average amplitude of +/−16 ms around the minimum, which was found at 256 ms. To quantify the P3, we estimated the maximal amplitude between 300 and 600 ms at Pz and extracted the average amplitude of +/−16 ms around the maximum, which was found at 360 ms (see Fig. 3). Additionally, for brain-behavior-relations, we also extracted the single-trial data in the same time frame and at the same electrodes.

### Statistical analyses

We used ANOVA and mixed model analyses for statistical analyses. For behavioral effects, an ANOVA was computed where the number of units of fish taken by the participants served as dependent variable, predicted by the factors *partner* (person versus computer) and *stakes* (money versus points). Additionally, trait greed was entered as covariate after z-standardization. Neural correlates of decision making were analyzed by an ANOVA with amplitude of the FRN as dependent variable, with the same independent variables and covariate and, additionally, the factor *feedback* (number of fish taken by the partner: 2 units of fish vs. more than 2 units of fish). To investigate brain-behavior relations, we pursued a single trial approach to predict the number of units of fish in trial n + 1 using a mixed model approach, with z-standardized P3 amplitude, z-standardized trait greed and the factor *feedback* (number of fish taken by the partner: 2 units of fish vs. more than 2 units of fish) as predictors and random slopes for the factors *partner* (person versus computer) and *stakes* (money versus points). All analyses were performed in RStudio 1.1.453 with the packages psych^41^ and lme4^42^.

## Data Availability

All data are available on https://osf.io/pjky9/.
